# Calcium Electroporation Reduces Viability and Proliferation Capacity of Four Uveal Melanoma Cell Lines in 2D and 3D Cultures

**DOI:** 10.3390/cancers14122889

**Published:** 2022-06-11

**Authors:** Miriam M. Kraemer, Theodora Tsimpaki, Utta Berchner-Pfannschmidt, Nikolaos E. Bechrakis, Berthold Seitz, Miltiadis Fiorentzis

**Affiliations:** 1 Department of Ophthalmology, University Hospital Essen, University of Duisburg-Essen, Hufeland Str. 55, 45147 Essen, Germany; miriam.kraemer@uk-essen.de (M.M.K.); theodora.tsimpaki@gmail.com (T.T.); utta.berchner-pfannschmidt@uk-essen.de (U.B.-P.); nikolaos.bechrakis@uk-essen.de (N.E.B.); 2 Department of Ophthalmology, Saarland University Medical Center, Kirrberger Str. 100, 66421 Homburg, Germany; berthold.seitz@uks.eu

**Keywords:** uveal melanoma, calcium electroporation, electrochemotherapy, bleomycin, 3D tumor spheroids, cytotoxic effects, long-time survival, anti-tumor potential

## Abstract

**Simple Summary:**

Calcium electroporation (CaEP) is an innovative anti-tumor treatment modality that induces cell death by introducing supraphysiological concentrations of calcium into cells with a limited effect on normal cells. The objective of the present study is to assess the effect of CaEP in uveal melanoma (UM) cell lines in comparison to electrochemotherapy (ECT) with bleomycin using 2D monolayer cell cultures as well as 3D tumor spheroid models in four different UM cell lines. The morphological changes of the spheroids, the cell viability, growth rate as well as the cytotoxic effect of electroporation (EP) with calcium chloride and bleomycin were evaluated with various drug concentrations. The results of CaEP and ECT both suggest a comparable dose-dependent reduction in cell viability and proliferation rate in all tested 2D cell lines and 3D tumor spheroids. These data point out that CaEP is an established anticancer treatment causing cell death by ATP depletion in in vitro and in vivo, representing an efficient alternative therapy with a lower cytotoxic potency for the local UM tumor control.

**Abstract:**

Electrochemotherapy (ECT) is the combination of transient pore formation following electric pulse application with the administration of cytotoxic drugs, which enhances the cytotoxic effect of the applied agent due to membrane changes and permeabilization. Although EP represents an established therapeutic option for solid malignancies, recent advances shift to the investigation of non-cytotoxic agents, such as calcium, which can also induce cell death. The present study aims to evaluate the cytotoxic effect, the morphological changes in tumor spheroids, the effect on the cell viability, and the cell-specific growth rate following calcium electroporation (CaEP) in uveal melanoma (UM) 2D monolayer cell cultures as well as in 3D tumor spheroid models. The experiments were conducted in four cell lines, UM92.1, Mel270, and two primary UM cell lines, UPMD2 and UPMM3 (UPM). The 2D and 3D UM cell cultures were electroporated with eight rectangular pulses (100 µs pulse duration, 5 Hz repetition frequency) of a 1000 V/cm pulse strength alone or in combination with 0.11 mg/mL, 0.28 mg/mL, 0.55 mg/mL or 1.11 mg/mL calcium chloride or 1.0 µg/mL or 2.5 µg/mL bleomycin. The application of calcium chloride alone induced an ATP reduction only in the UM92.1 2D cell cultures. Calcium alone had no significant effect on ATP levels in all four UM spheroids. A significant decrease in the intracellular adenosine triphosphate (ATP) level was documented in all four 2D and 3D cell cultures for both CaEP as well as ECT with bleomycin. The results suggest a dose-dependent ATP depletion with a wide range of sensitivity among the tested UM cell lines, control groups, and the applied settings in both 2D monolayer cell cultures and 3D tumor spheroid models. The colony formation capacity of the cell lines after two weeks reduced significantly after CaEP only with 0.5 mg/mL and 1.1 mg/mL, whereas the same effect could be achieved with both applied bleomycin concentrations, 1.0 µg/mL and 2.5 µg/mL, for the ECT group. The specific growth rate on day 7 following CaEP was significantly reduced in UM92.1 cell lines with 0.5 and 1.1 mg/mL calcium chloride, while Mel270 showed a similar effect only after administration of 1.1 mg/mL. UM92.1 and Mel270 spheroids exhibited lower adhesion and density after CaEP on day three in comparison to UPM spheroids showing detachment after day 7 following treatment. CaEP and bleomycin electroporation significantly reduce cell viability at similar applied voltage settings. CaEP may be a feasible and inexpensive therapeutic option for the local tumor control with fewer side effects, in comparison to other chemotherapeutic agents, for the treatment of uveal melanoma. The limited effect on normal cells and the surrounding tissue has already been investigated, but further research is necessary to clarify the effect on the surrounding tissue and to facilitate its application in a clinical setting for the eye.

## 1. Introduction

Uveal melanoma (UM) is a relatively rare disease with an incidence of approx. five cases per million in the USA and approx. 1–8 cases per million in Europe per year, however, constituting the most common primary intraocular tumor in adults [1,2]. It arises from neoplastic melanocytes along the uveal tract, most commonly occurring in the choroid (approx. 85%) and the ciliary body or the iris [1,2,3,4]. While several prognostic genetic changes have been identified in UM, epigenetic influences are gradually intensely emphasized [1,3,5,6,7]. Further risk factors are the presence of light eyes or fair complexion, iris or choroidal nevus, ocular or oculodermal melanocytosis as well as dysplastic nevus syndrome [4,8].

Furthermore, the molecular pathogenesis of UM is characterized by chromosomal abnormalities and gene mutations. Most common are mutations in the guanine nucleotide-binding protein G(q) subunit alpha (GNAQ) and the guanine nucleotide-binding protein subunit alpha-11 (GNAQ/GNA11), which occur in a mutually exclusive manner in approx. 90% of posterior UMs [7]. Other commonly detected mutations in UM are the BRCA1-associated protein-1 (BAP1), splicing factor 3B subunit 1 (SF3B1), eukaryotic translation initiation factor 1A, X-chromosomal (EIF1AX) as well as telomerase reverse transcriptase promoter (TERTp). Moreover, recent advancements in the molecular pathophysiology improved the understanding of signaling pathways and their role in the development of UM, such as MEK, the retinoblastoma (Rb) pathway, p53 signaling, the P13K/AKT, and MAPK pathway, as well as PTEN, GNAQ/11, andBAP1 mutations [3,9]. The most common chromosomal abnormalities include loss of the 1p, 3, 6q, and 8p chromosomes as well as a gain of the 1q, 6p, and 8q chromosomes. Monosomy 3 is present in 65% of UM and is associated with a particularly poor prognosis [4,5,7,10,11].

Treatment options range from local therapy, including radiotherapy (brachytherapy or teletherapy), thermotherapy, and tumor resection (transscleral resection, and endoresection) to enucleation [12]. The prognosis is associated with different variables, including the location and size of the tumor, age of the patient, the extent of a possible metastasis but foremost the histological characteristics, tumor cytology, circulating cells, and specific gene profiling. Uveal melanoma has a high tendency to metastasize (approximately 50%) within 10 years after the first diagnosis irrespectively from the type of treatment, resulting in high mortality [13,14,15].

Despite advancements in the management of localized disease, adjuvant therapy of metastatic disease in the form of chemotherapy, adjuvant immunotherapy, and molecular targeted therapy may be necessary to prevent further progression, prolong life expectancy, and improve quality of life. The research and establishment of additional globe-preserving treatment strategies are, therefore, crucial for uveal melanoma patients [16].

Electroporation-based techniques, such as irreversible electroporation, electrochemotherapy, and gene therapy, have already been established as effective alternative treatment modalities, especially for inoperable, recurring, and chemo-resistant malignancies in various tumor entities that do not adequately respond to the current treatment regimen. Electroporation (EP) is the induction of transient permeabilization of the cell membrane via short high voltage electric pulses, allowing passage of ions and molecules into and out of the cell. Electrochemotherapy is emerging (ECT) as an adjuvant treatment modality for local tumor control in various cancer entities. It combines the application of electric pulses to the tumor tissue with the administration of chemotherapeutic agents, rendering the cell membrane permeable to otherwise impermeant or poorly permeant anticancer drugs [17,18,19,20,21,22,23,24,25,26,27].

Calcium electroporation (CaEP) is a prospective anticancer treatment where high concentrations of calcium are introduced into the cytosol following the application of electric pulses. The high intracellular concentrations of calcium ions can lead to an adenosine triphosphate (ATP) deficit [28,29,30,31]. The ATP depletion may be attributed to various pathomechanisms, such as direct loss of ATP through the permeabilized membrane, increased consumption of ATP by the Ca^2+^-ATPases to transport calcium out of the cells, and reduced production of ATP due to disruption in the mitochondrial membrane potential by the high intracellular calcium concentrations [32,33,34,35].

Preclinical investigations suggest that this acute and severe ATP depletion leads to necrotic, apoptotic, or autophagic cell death [28,29,30,31]. Using the ESOPE protocol, both ECT and CaEP show comparable results regarding tumor control and cell viability in different tumor types [36,37,38]. CaEP offers several advantages, including being inexpensive, non-mutagenic, and having long durability [39]. The present study aims to evaluate the cytotoxic effect, the morphological changes, the effect on the cell viability, as well as on the cell-specific growth rate following CaEP and ECT in UM 2D monolayer cell cultures and 3D spheroid models in four UM cell lines.

## 2. Materials and Methods

### 2.1. Characterization of Uveal Melanoma Cells Lines

To reproduce the heterogenic characteristics of uveal melanoma, four UM cell lines with different genetic profiles were analyzed in the present study: two UM cell lines, UM92.1 and Mel270, and two uveal primary melanoma (UPM) cell lines, UPMD2 and UPMM3. The genetic and morphologic characteristics are summarized in Table 1 [27].

All cell lines were authenticated by short tandem repeat profiling according to published data [21,40,41,42,43,44].

**Table 1:** Characteristics of uveal melanoma cell lines.

Cell Line	Genetics	Morphology/Doubling Time	References
UM92.1	GNAQ Q209L, Disomy-3, WT BAP1, EIF1AX	Epithelioid/38–58 h	[40,41,42,43]
Mel270	GNAQ Q209P, Disomy-3, WT BAP1	Spindle/43 h	[41,42,44]
UPMD2	GNA11 Q209L, Disomy-3, WT BAP1	Epithelioid/150 h	[21,45]
UPMM3	GNAQ Q209P, Monosomy-3, Mutant BAP1	Spindle and epithelioid/100 h	[45]

### 2.2. 2D and 3D Cultures of Uveal Melanoma Cell Lines

The UM cell lines UM92.1 and Mel270 were maintained in RPMI 1640 medium (GIBCO, Fisher Scientific, Thermo Fisher Scientific Inc., Waltham, MA, USA). The UPM cell lines UPMD2 and UPMM3 were cultivated in Ham/F12 medium (PAN-Biotech GmbH, Aidenbach, Germany). The cell medium was supplemented with 10% fetal calf serum and 1% penicillin-streptomycin (5000 U/mL) and was exchanged two times per week. The cell cultures were incubated in a humidified incubator (37 °C, 5% CO_2_).

The 3D spheroids were generated by seeding 5 × 10^3^ cells/well in 96-well ultra-low attachment plates (PHC Corporation, Tokyo, Japan) in 100 µL of the respective cell culture medium. The spheroid cultures were incubated in a humidified chamber (37 °C, 5% CO_2_) for one week until electroporation.

### 2.3. Electroporation of 2D and 3D Uveal Melanoma Cell Cultures

The UM cells were trypsinized and transferred into Ingenio cuvettes (Mirus Bio LLC, Madison, WI, USA) for the formation of monolayer cell cultures. A total of 5 × 10^5^ cells were resuspended in 400 µL Hepes buffer containing 0, 0.11 mg/mL, 0.28 mg/mL, 0.55 mg/mL or 1.11 mg/mL calcium chloride or 1.0 µg/mL or 2.5 µg/mL bleomycin.

Seven days after generating the UM spheroids, the medium was gently removed and replaced with 100 µL Hepes buffer containing 0, 0.55 mg/mL, 1.11 mg/mL, 2.78 mg/mL, 5.55 mg/mL, and 8.33 mg/mL calcium chloride or 1.0 µg/mL or 2.5 µg/mL bleomycin. The spheroids were treated at higher concentrations of calcium chloride because preliminary tests showed no effect after treatment at the concentrations used on the single-cell model (data not shown).

The monolayer cell cultures and the spheroids were then electroporated using a Genedrive voltage pulse generator (IGEA S.p.A., Carpi, Italy) with an electroporator adapter for cuvettes and a two parallel aluminum electrode (4 mm apart) respectively. In this study, cells and spheroids were electroporated with eight rectangular pulses (100 µs pulse duration, 5 Hz repetition frequency) of a 1000 V/cm pulse strength according to the European Standard Operating Procedure on Electrochemotherapy (ESOPE) alone or in combination with the aforementioned calcium and bleomycin concentrations.

### 2.4. Analysis of Cellular ATP Level Following CaEP and ECT in 2D and 3D Uveal Melanoma Cell Cultures

Following electroporation of monolayer cell cultures, the cell suspensions, approx. 10^4^ cells were transferred to white opaque-walled multi-well plates (Nunc F96 Microwell Polysterolplate white, Thermo Fisher Scientific, Roskilde, Denmark) in 100 µL medium (RPMI 1640) or Ham/F12 medium, supplemented with 10% fetal calf serum and 1% penicillin-streptomycin (5000 U/mL) per well. Changes in cellular ATP concentrations (N = 3 or 4; *n* = 8 each condition) were analyzed using the CellTiter-Glo 2.0 assay (Promega GmbH, Walldorf, Germany) according to the manufacturer’s instructions. Equal portions of CellTiter-Glo 2.0 and cell suspension were mixed and placed in the centrifuge (750 rpm) for two minutes. After a further 15 min of incubation in a dark chamber at room temperature, the luminescence was recorded using the plate reader ClarioStar Plus (BMG LABTECH, Ortenberg, Germany). The ATP luminescence was given in RLU (relative light units).

The cellular ATP level in UM and UPM spheroids (N = 3 or 4, *n* = 5–8 each condition) was assessed using the CellTiter-Glo 3D (Promega) according to the manufacturer’s instructions. Equal amounts of CellTiter-Glo 3D and spheroid cultures were mixed by pipetting up and down for 30 s to ensure complete lysis of the cells and the release of ATP. The mixture was transferred to white opaque-walled multi-well plates. After five minutes of incubation in the centrifuge (750 rpm) and a further 25 min in a dark chamber at room temperature, the luminescence was measured using a reader ClarioStar Plus. The ATP luminescence was given in RLU.

### 2.5. Cell Viability and Cell-Specific Growth Rate in 2D Cell Cultures Following CaEP and ECT

After the electroporation procedure, the UM and UPM cells (*n* = 3 or 4; *n* = 8 each condition) were seeded on flat bottomed 96-well plates (Sarstedt, Nürnbrecht, Germany) with 10^4^ cells in 100 µL medium per well. To determine cell viability and growth, the cells were fixated 24 h, 3 days, and 7 days after electroporation with 50 µL 10% trichloroacetic acid (TCA, in Aqua dest.; Fluka, Honeywell International Inc., Charlotte, NC, USA) for one hour at 4 °C and then stained with 50 µL 0.4% Sulforhodamine B solution (SRB, in 1% acetic acid; Sigma-Aldrich, Merck KGaA, Darmstadt, Germany). After approximately three washing steps with 1% acetic acid, the minoxanthene dye was then dissolved with 150 µL 10 mM Tris buffer (pH 10.5). Finally, the absorbance was recorded at 560 nm using the reader ClarioStar Plus (BMG LABTECH GmbH, Ortenberg, Germany). Based on these data, the cell line-specific growth rate (SGR) in % per day was calculated by using the following Equation (1).

(1) $$\mathrm{SGR}=\frac{\ln\left(\mathrm{Nt}/N0\right)}{t2-t0\;}\times 100\%$$

N = absorbance units; t = time point.

### 2.6. Morphology and Growth of 3D Spheroids Following CaEP and ECT

The image documentation of the 3D multicellular spheroids was conducted before (d0), on day 3 (3 d), as well as on day 7 (7 d) after treatment using a Zeiss Primovert bright-field microscope at 4× magnification. The image and morphological analysis of the spheroids was carried out with a Zeiss Axiocam 105 and ZENcore software (Carl Zeiss AG, Oberkochen, Germany). The optic density (mean gray value; mgv) and the cross-sectional area were measured to evaluate the mass density of the spheroids. Due to frequent detachment of cells from the main spheroid core due to low adhesion and density following treatment, the Feret’s diameter was calculated. This value represents the mean diameter of the not dissociated spheroid as well as the mean distance between the most distant spheroid compartments. All measurements were performed using the open-source software Fiji ImageJ 1.53c (MPI-CBG, Dresden, Germany).

### 2.7. Colony Formation Following CaEP and ECT in 2D and 3D UM Cell Cultures

UM and UPM cell suspensions of the monolayer cell cultures (*n* = 3–6 each condition) were diluted in medium (RPMI or Ham’s F12) supplemented with 10% fetal calf serum and 1% penicillin-streptomycin (5000 U/mL) and seeded on Cellstar 6-well plates (Greiner Bio-One GmbH, Frickenhausen, Germany) with 2 × 10^3^ cells in 3 mL medium per well.

One week after the electroporation procedure, spheroids treated the same were pooled and seeded on 6-well plates (Cellstar, Greiner Bio-One GmbH, Frickenhausen, Germany) with 2 × 10^3^ cells per well and incubated in a humidified chamber at 37 °C with 5% CO_2_. The medium was exchanged once a week. After an incubation period of 3 weeks, the medium was removed, and the spheroids were dissociated by pipetting and pooling. Finally, the cells were plated on a Cellstar 6-well plate (Greiner Bio-One GmbH, Frickenhausen, Germany) with 2 × 10^3^ cells in 3 mL medium per well.

After a two-week for monolayer and a three-week incubation phase for spheroids in a humidified chamber (37 °C, 5% CO_2_), the medium was removed, and the cells were stained with 4% crystal violet solution. To assess the survival fraction (SF) of proliferating cancer cells, colonies containing 50 or more cells were counted using a Leica S6D Greenough stereo microscope (Leica microsystems GmbH, Wetzlar, Germany). The plating efficiency (PE; Equation (2)) and, therefore, the survival fraction (SF; Equation (3)) were calculated in percentage and statistically analyzed.

(2) $$\mathrm{PE}=\;\frac{\mathrm{no}.\;\mathrm{of}\;\mathrm{colonies}\;\mathrm{formed}}{\mathrm{no}.\;\mathrm{of}\;\mathrm{cells}\;\mathrm{seeded}\;}\;\times 100\%$$

(3) $$\mathrm{SF}\;=\;\frac{\mathrm{no}.\;\mathrm{of}\;\mathrm{colonies}\;\mathrm{formed}\;\mathrm{after}\;\mathrm{treatment}}{\mathrm{no}.\;\mathrm{of}\;\mathrm{cells}\;\mathrm{seeded}\;\times \;\mathrm{PE}}\;\times 100\%\;$$

### 2.8. Statistical Analysis

Statistical analysis of the data was performed using a two-way ANOVA and Tukey’s multiple comparisons test (GraphPad Prism 9.20 software, GraphPad Software Inc., San Diego, CA, USA). A value of *p* < 0.05 was considered statistically significant and significance levels were indicated with * *p* < 0.05, ** *p* < 0.01, *** *p* < 0.001, **** *p* < 0.0001.

## 3. Results

### 3.1. Effects of CaEP and ECT on ATP Levels

**Figure 1:**
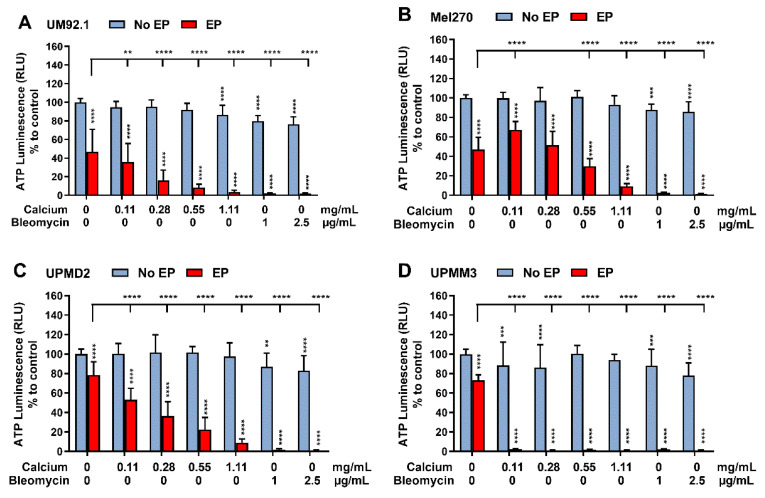
Intracellular ATP level in 2D UM cell cultures after CaEP and ECT. ATP depletion after calcium electroporation (0.11, 0.28, 0.55 or 1.11 mg/mL) compared to ECT with bleomycin (1 or 2.5 μg/mL) on (**A**) UM92.1, (**B**) Mel270, (**C**) UPMD2, and (**D**) UPMM3 single-cell suspension (N = 3 or 4; *n* = 8 each condition) was assessed by lysing the cells and measuring the ATP luminescence (Relative Light Units; RLU) 24 h after treatment. The analyses show the non-electroporated (no EP) and the electroporated groups (EP) alone or in combination with the tested calcium and bleomycin concentrations. The EP was conducted with 1000 V/cm pulse strength, 100 µs pulse duration, and 5 Hz repetition frequency. Untreated cells, calcium chloride alone, bleomycin alone (no EP group), or EP alone constitute the control groups. Statistical analysis was performed using a two-way ANOVA and Tukey’s multiple comparisons test. Statistically significant levels are indicated with * *p* < 0.05, ** *p* < 0.01, *** *p* < 0.001, **** *p* < 0.0001.

**Figure 2:**
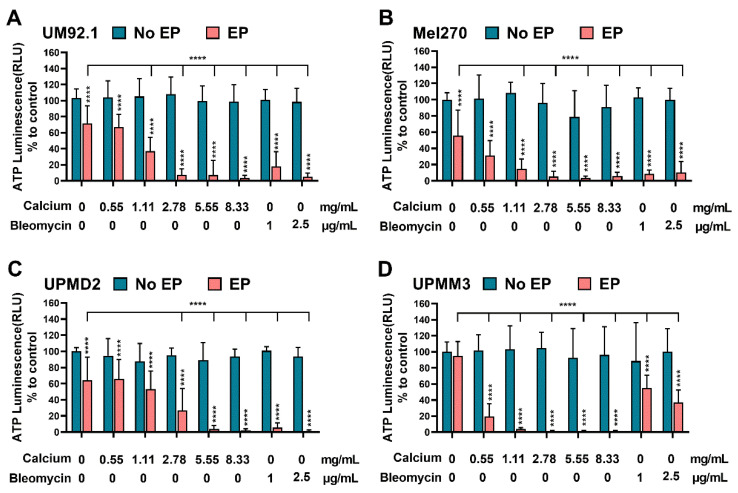
Effect of CaEP and ECT on UM and UPM tumor spheroid ATP level. (**A**) UM92.1 spheroids, (**B**) Mel270 spheroids, (**C**) UPMD2 spheroids, and (**D**) UPMM3 spheroids were incubated with different concentrations of calcium chloride (0.55, 1.11, 2.78, 5.55, or 8.33 mg/mL) or bleomycin (1 μg/mL or 2.5 μg/mL) and electroporated (1000 V/cm pulse strength, 100 µs pulse duration and 5 Hz repetition frequency; EP group). Untreated cells, calcium chloride alone, bleomycin alone (no EP group) or EP alone constitute the control groups. Statistical analysis was performed using a two-way ANOVA and Tukey’s multiple comparisons test. Statistically significant levels are indicated with * *p* < 0.05, ** *p* < 0.01, *** *p* < 0.001, **** *p* < 0.0001.

To assess the cytotoxicity of CaEP compared to ECT with bleomycin in UM and UPM cells and spheroids, an ATP assay was performed 24 h after treatment (Figure 1 and Figure 2). The analysis shows a significant ATP depletion after treatment with 1 μg/mL as well as 2.5 μg/mL bleomycin even without the application of electrical pulses in all analyzed 2D cell cultures compared to the untreated control group (Figure 1A–D). However, exposure to the indicated calcium chloride concentrations without application of electrical pulses had no significant impact on cellular ATP levels except for the UM92.1 cell line after administration of 1.11 mg/mL. EP alone also significantly reduced the ATP levels in UM92.1. Overall, all lines showed a highly significant decrease in ATP compared to both control groups following EP with calcium and bleomycin, accompanied by slight differences in sensitivity. A higher ATP depletion was achieved in the Mel270 following EP alone in comparison with CaEP with a concentration of 1.11 mg/mL. The UPMM3 cell line shows a higher depletion of ATP after administration of 0.11 and 0.28 mg/mL calcium chloride alone with respect to the other cell lines, as well as a more dramatic reduction in ATP after the combination of low calcium concentrations with EP (Figure 1D). After EP with increasing concentrations of calcium chloride (0.11; 0.28; 0.55; 1.11 mg/mL), a dose-dependent and significant ATP depletion was documented in all tested cell lines and in comparison to the control groups resulting in outcomes similar to ECT.

The ATP levels of UM and UPM spheroids were only marginally affected following calcium chloride and bleomycin administration without EP (Figure 2A–D). Electroporation alone reduced the ATP levels significantly in UM92.1, Mel270, and UPMD2 spheroids (Figure 2A–C). UPMM3 did not respond after the application of EP alone but showed a higher reduction in ATP in comparison to the tested cell lines and control groups in the CaEP group, even with low calcium concentrations. A higher sensitivity of UPMD2 spheroids was noted in the ECT group. All tested UM spheroids exhibit a statistically significant reduction in ATP for the tested condition of ECT and CaEP.

### 3.2. Effects of CaEP and ECT on Cell and Spheroid Growth

**Figure 3:**
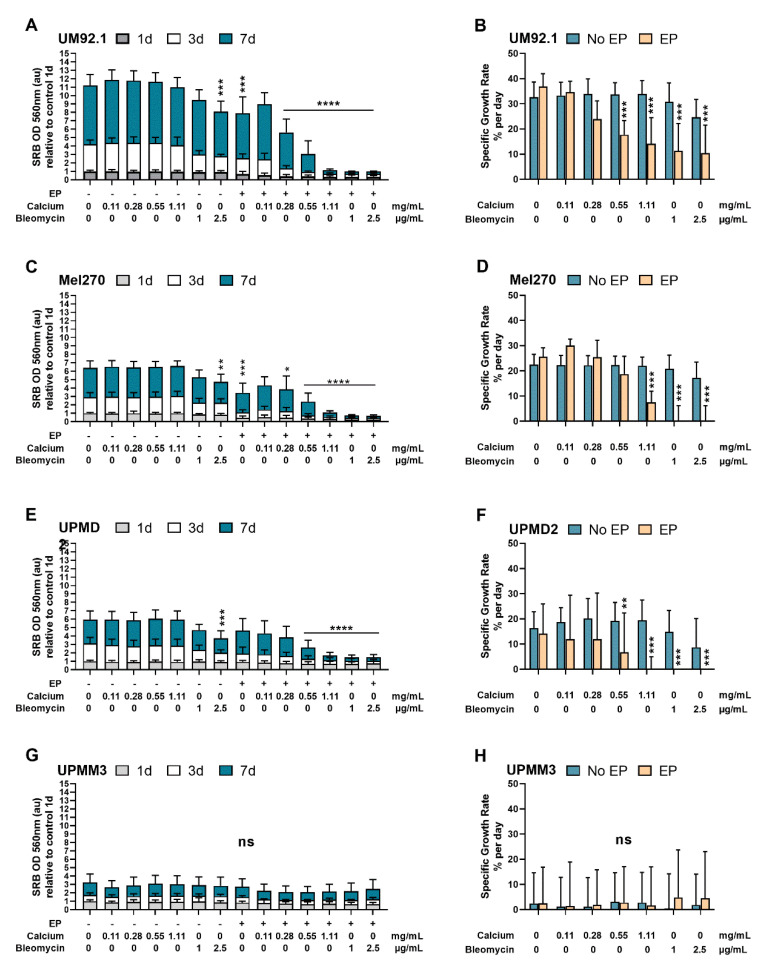
Effects of CaEP and ECT on cell viability and cell growth. The different UM cell lines. (**A**,**B**) UM92.1, (**C**,**D**) Mel270, (**E**,**F**) UPMD2, and (**G**,**H**) UPMM3 were exposed to calcium chloride (0.11, 0.28, 0.55 or 1.11 mg/mL) or bleomycin (1 or 2.5 μg/mL). EP was conducted with 1000 V/cm pulse strength, 100 µs pulse duration, and 5 Hz repetition frequency. Untreated cells, calcium chloride alone, bleomycin alone (no EP group), or EP alone constitute the control groups. The cell count and, therefore, the cytotoxic effect (N = 3–4; *n* = 8 each condition) were assessed by measuring SRB absorbance values at 560 nm cell cultures stained with SRB on day 1 (1 d), 3 (3 d), and 7 (7 d) after treatment (**A**,**C**,**E**,**G**). The data are shown as absorbance units (au) (mean values ± SD). Cell line-specific growth rates in percentage per day (mean ± SD) were calculated and statistically compared to the control groups (**B**,**D**,**F**,**H**). Statistical analysis was performed using a two-way ANOVA and Tukey’s multiple comparisons test.; significance levels are indicated with * *p* < 0.05, ** *p* < 0.01, *** *p* < 0.001, **** *p* < 0.0001.

The sulforhodamine B (SRB) assay was used for cell density determination and, therefore, as a cytotoxicity screening of the tested agents. The tumor growth was quantified by calculating the specific growth rate (SGR). The cytotoxic effect of 2.5 µg/mL bleomycin and EP alone in the UM92.1 (Figure 3A) and Mel270 (Figure 3B) cells was statistically significant on day 7 and higher than following CaEP with 0.11 for both cell lines. Calcium alone did not exhibit a significant reduction in cell count in contrast to bleomycin alone. UPMD2 illustrates (Figure 3E,F) a significant decrease in cell viability on day 7 after treatment only with 2.5 mg/mL bleomycin when no electric voltage was applied (*p* < 0.005). The reduction in cell viability and the cytotoxic effect of the tested conditions were not statistically significant in the UPMM3 cells (Figure 3G). Nevertheless, a decrease in the cell viability on day one after ECT with both tested concentrations as well as after CaEP with 0.28 mg/mL up to 1.11 mg/mL calcium chloride was noted.

One week following CaEP, a significant reduction in the cell viability with a mean specific growth rate (Figure 3B) of 17.71% per day (0.55 mg/mL calcium) and 14.09% per day (1.11 mg/mL calcium) was documented. After ECT with bleomycin, the UM92.1 cells showed a comparable mean specific growth rate of 11.26% (1 μg/mL) and 10.45% per day (2.5 μg/mL) and a statistically significant reduction in cell viability. Similar results could be achieved for Mel270 cells only after CaEP with concentrations of 1.1 mg/mL of calcium chloride (Figure 3C,D). The specific growth rate was significantly affected in Mel270 cells after CaEP with 1.11 mg/mL calcium (mean of 7.45% per day) and after ECT with both concentrations (mean of 0% per day). In the UPMD2 cells, the mean specific growth rate was calculated 6.75% per day after CaEP with 0.55 mg/mL calcium chloride and 0% per day after CaEP/ECT with 1.11 mg/mL calcium or 1 μg/mL and 2.5 μg/mL bleomycin, respectively. Significant differences in the specific growth rates between the treated and untreated cohorts could not be observed in the UPMM3 cell line (Figure 3H).

**Figure 4:**
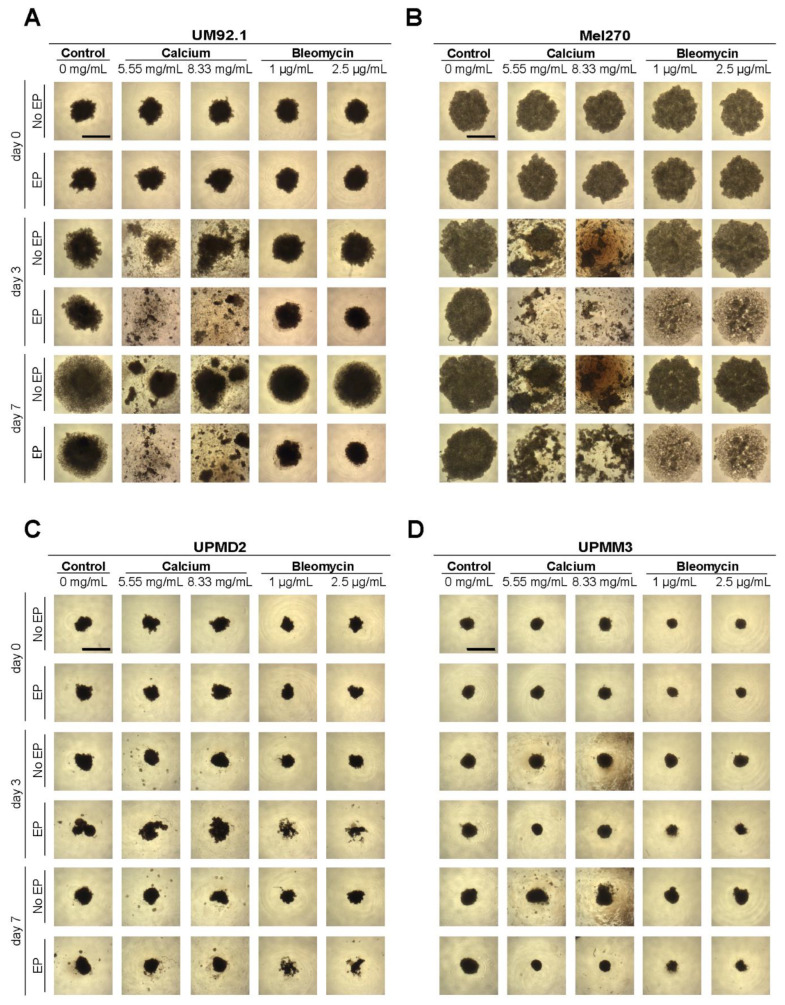
UM and UPM spheroids after EP with calcium chloride and bleomycin. Image documentation of (**A**) UM92.1, (**B**) Mel270, (**C**) UPMD2, and (**D**) UPMM3 spheroids was conducted before (day 0) and on days 3 and 7 after electroporation (EP) in combination with calcium chloride (5.55 or 8.33 mg/mL) or bleomycin (1 or 2.5 μg/mL). Untreated cells, calcium chloride alone, bleomycin alone (no EP group), or EP alone constitute the control groups. Images were recorded at 4× magnification; scale bars represent 100 µm.

**Figure 5:**
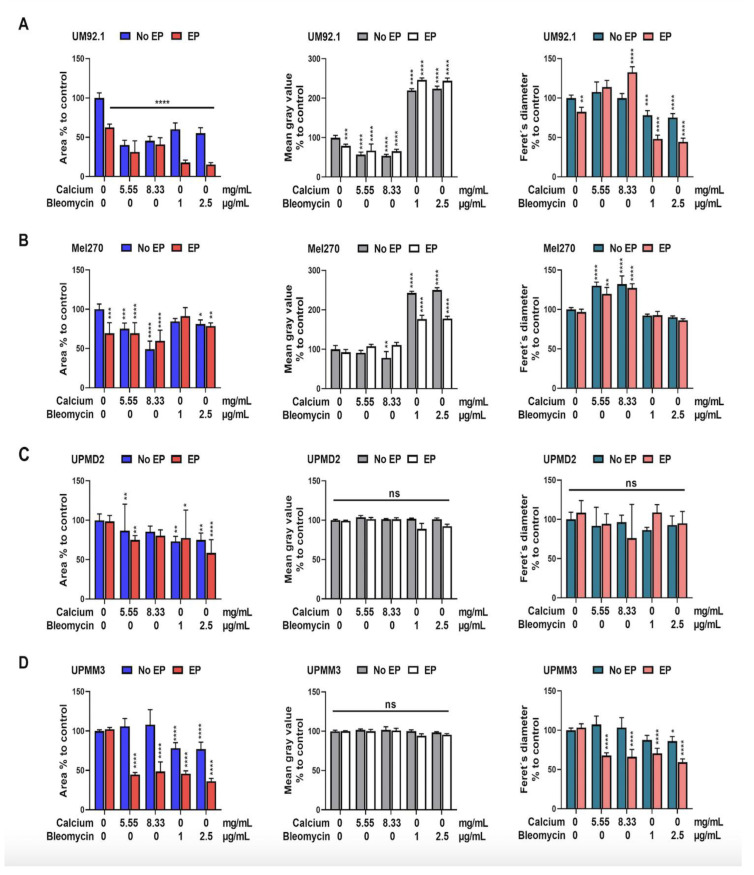
Cross-sectional area, mean gray value, and Feret’s diameter of UM and UPM spheroids on day 7 following EP in combination with calcium chloride or bleomycin. (**A**) UM92.1, (**B**) Mel270, (**C**) UPMD2, and (**D**) UPMM3 spheroids were incubated with different concentrations of calcium chloride (0.55, 1.11, 2.78, 5.55, or 8.33 mg/mL) or bleomycin (1 μg/mL or 2.5 μg/mL) and electroporated (1000 V/cm pulse strength, 100 µs pulse duration and 5 Hz repetition frequency; red (EP). Untreated cells, calcium chloride alone, bleomycin alone (no EP group), or EP alone constitute the control groups. To assess spheroid density, mass, expansion/shrinkage, the area (left column), the optic density (mean gray value; middle column), and the mean Feret´s diameter area (right column) of the detected spheroid or spheroid compartments were calculated (*n* = 4–5 each condition). Statistical analysis was performed using a two-way ANOVA and Tukey’s multiple comparisons test. Significance levels are indicated with * *p* < 0.05, ** *p* < 0.01, *** *p* < 0.001, **** *p* < 0.0001.

The image documentation was conducted before and on days 3 and 7 following treatment (Figure 4). The mass, spread or shrinkage, and the cell density of the spheroids were quantitatively measured using the mean gray value (mgv), the calculation of the cross-sectional area, and the Feret’s diameter (Figure 5). The evaluation of the spheroid images shows a dramatic reduction in size, especially in the UM92.1 and Mel270 cell lines, accompanied by low adhesion and peripheral cell distribution. The Feret’s diameter shows a statistically significant increase in UM92.1 and Mel 270 and a decrease in UPMM3 after CaEP with 8.33 mg/mL. On the other hand, ECT led to a significant decrease in Feret’s diameter in UM92.1 and UPMM3 cell lines. The effect of CaEP on the reduction in the cross-sectional area was more apparent in the Mel 270 cells with or without EP in comparison with ECT. CaEP and ECT had a similar effect on the cross-sectional area in all cell lines, with the exception of UM92.1, where the cells exhibited a more drastic response following ECT (Figure 5). An increase in the mean gray value was observed foremost in the UM92.1 and Mel270 cell lines after ECT, whereas a statistically significant decrease in the mean gray value and the cell density was documented after CaEP with 8.33 mg/mL. In the UPMD2 and UPMM3 cell lines, the mean gray value remained stable in all cohorts.

### 3.3. Effect of CaEP and ECT on the Colony Formation Capacity of 2D and 3D UM and UPM Cell Cultures

**Figure 6:**
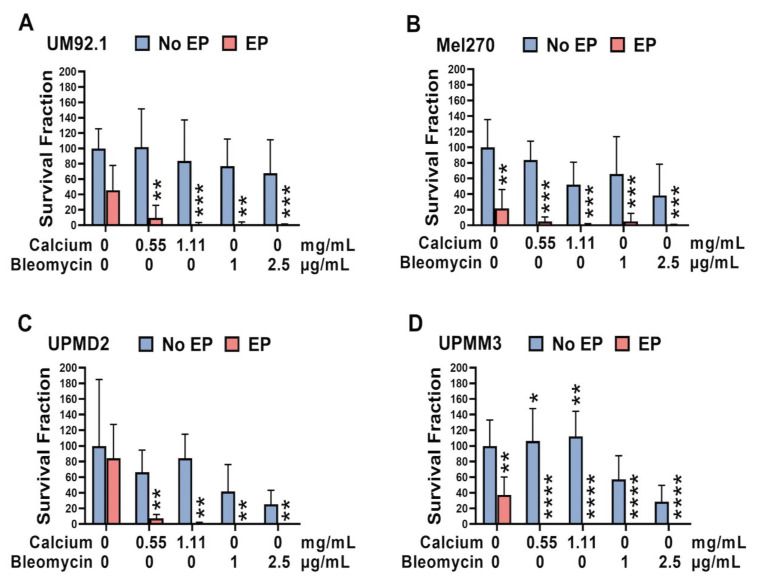
Survival fraction of UM and UPM cells following CaEP and ECT. To investigate the effect of CaEP and ECT on the cell growth of 2D UM and UPM cell cultures, the fraction of seeded cells that retained colony formation capacity was calculated two weeks after treatment (*n* = 3–6 each condition). Therefore, (**A**) UM92.1, (**B**) Mel270, (**C**) UPMD2, and (**D**) UPMM3 cell suspensions were treated with calcium chloride (0.11, 0.28, 0.55, or 1.11 mg/mL) or bleomycin (1 or 2.5 μg/mL) and electroporated with 1000 V/cm pulse strength, 100 µs pulse duration, and 5 Hz repetition frequency. Untreated cells, calcium chloride alone, bleomycin alone (no EP group), or EP alone constitute the control groups. To determine the survival fraction, colonies were stained with a 4% crystal violet solution. For the present analysis, only colonies consisting of at least 50 cells were included. Based on the amount of counted colonies, the cell-specific plating PE and the treatment-specific SF were calculated. The statistical analysis was performed using a two-way ANOVA and Tukey’s multiple comparisons test. The data are presented as mean values ± SD; significance levels are indicated with * *p* < 0.05, ** *p* < 0.01, *** *p* < 0.001, **** *p* < 0.0001.

The survival fraction in the monolayer cell lines was significantly decreased in all tested cell lines for all tested conditions (Figure 6). A statistically significant increase in SF was only documented in the UPMM3 cells after administration of calcium or bleomycin alone. EP alone induced an apparent reduction in the survival fraction in the Mel270 and UPMM3 cell lines (Figure 6B,D).

**Figure 7:**
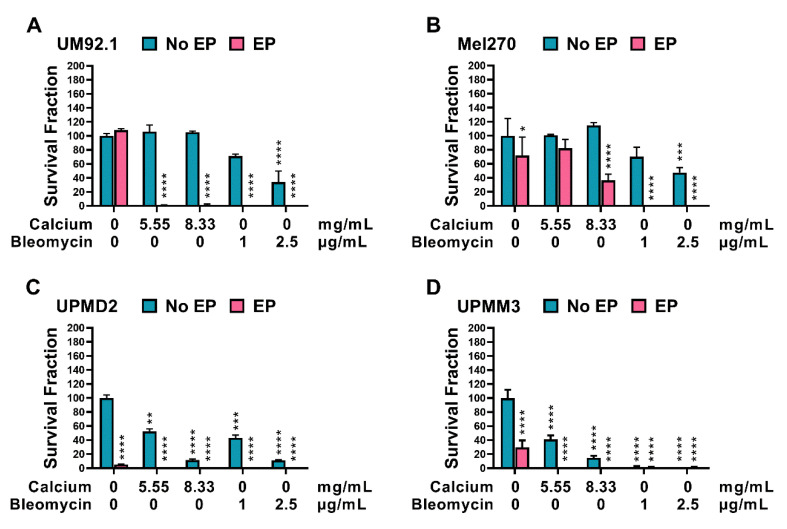
Survival fraction of UM and UPM spheroids following CaEP and ECT. (**A**) UM92.1, (**B**) Mel270, (**C**) UPMD2, and (**D**) UPMM3 spheroids were treated with 5.55 or 8.33 mg/mL of calcium chloride or with 1 μg/mL or 2.5 μg/mL of bleomycin in combination with EP (1000 V/cm pulse strength, 100 µs pulse duration and 5 Hz repetition frequency; EP group). Untreated cells, calcium chloride alone, bleomycin alone (no EP group), or EP alone constitute the control groups. After EP, spheroids were incubated for one week. After this incubation phase, the spheroids were dissociated, the cells were pooled and plated on a 6-well plate, and finally stained with 4% crystal violet solution. Only colonies consisting of at least 50 cells were counted. The statistical analyses were performed using a two-way ANOVA and Tukey’s multiple comparisons test. The data are presented as mean values ± SD; significance levels are indicated with * *p* < 0.05, ** *p* < 0.01, *** *p* < 0.001, **** *p* < 0.0001.

An overall reduction in the survival fraction and, therefore, the colony formation capacity of the tested spheroids was documented for all cell lines following CaEP and ECT (Figure 7A–D). The UPMM3 and UPMD2 cell lines showed a significant response and reduction in the survival fraction following the application of calcium and bleomycin alone, whereas no significant changes were observed in the no EP cohort group in the UM92.1 and Mel 270. A difference in the reduction in the survival fraction was noted between CaEP and ECT only in the Mel270 cells with a greater response following ECT, indicating a lower sensitivity to CaEP in comparison to the other cell lines. Interestingly a significant reduction was also illustrated in the UPMD2 and UPMM3 cell lines after EP alone (Figure 7C,D).

## 4. Discussion

ECT is a well-established electroporation-based treatment modality with high response rates in various tumor entities [36,37,38,46]. The electric pulses result in reversible cell poration with minimal ablative effects. However, the elementary mechanism of cell death following CaEP is dissimilar from ECT due to the absence of a chemotherapeutic agent. Apart from ATP depletion, an immunomodulatory effect, attributed to the reduction in suppressor cells and the increase in cytotoxic T lymphocyte activity, has been postulated [47]. Moreover, CaEP causes intratumorally vascular damage, resulting in tumor necrosis. Gibot et al. showed that the cell death following CaEP may also arise from mitochondrial dysfunction, without accompanying DNA damages, underlying its safety as a treatment option [48]. Intracellularly, calcium ion (Ca^2+^) plays an important role as a second messenger by maintaining cytoplasmic Ca^2+^ concentration low at rest and by mobilizing Ca^2+^ in response to stimulus, which in turn activates the cellular reaction [49]. In addition, calcium signaling regulates numerous basic cellular and biochemical processes, including cell proliferation and differentiation, and exhibits regulatory effects on various enzymes and proteins. Calcium levels must strictly remain at very low concentrations intracellularly via its removal to the extracellular environment and sequestration in the endoplasmic reticulum, maintaining the equilibrium of cytoplasmic Ca^2+^ level in concentrations about 20 to 40 nM [50].

Despite positive results in vitro and in vivo, the application of CaEP is mainly restricted to research purposes [51,52,53,54]. The applicability of CaEP for the treatment of UM has not yet been investigated. The present study aims to evaluate the cytotoxic effect, the morphological changes in tumor spheroids, the effect on cell viability, and cell-specific growth following CaEP and ECT in UM 2D monolayer cell cultures, as well as in 3D tumor spheroids. A dose-dependent reduction in ATP levels, cell viability as well as proliferation capacity with varying sensitivity was documented for all four tested UM cell lines following CaEP. ECT and CaEP showed a statistically significant reduction in ATP in all four tested cell lines in 2D and 3D tumor models. Higher sensitivity for CaEP regarding the ATP depletion was noted in both cell models for the UPMM3 cell line, exhibiting an apparent difference in comparison to ECT foremost in the 3D cell cultures. The great response following CaEP or ECT in the UPMM3 cells could not be reproduced with reference to the cell viability and the specific growth rate, which were not significantly affected after treatment. These results in the 2D cell cultures are in accordance with the minor morphological changes of the UPMM3 spheroids in size and cell distribution, as seen in Figure 5. In addition, a significant reduction in the cross-sectional area and the Feret’s diameter without alterations in the optic density and cell cohesion was detected in the UPMM3 spheroids. The discrepancy in the results between ATP levels, cell viability, and growth rate could be attributed to the doubling time of this cell line. Another explanation for the treatment resistance in the UPMM3 cell line could lie within the presence of monosomy 3, which is associated with a higher progression rate and therapy resistance [5,7,10,11,41,55]. Similarly, the UPMD2 cells also exhibit a lower sensitivity in comparison to the other UM-tested cell lines, thus showing a significant reduction in cell viability and specific growth rate following CaEP with a concentration of calcium chloride of 0.55 mg/mL or higher. The UPMD2 spheroids show more prominent morphologic changes after ECT in comparison to CaEP, whereas the optic density and Feret’s diameter remain non-significant for both treatment modalities, underlying the importance of image documentation and automatized image analysis for the proper interpretation of the results.

UM92.1 cells show a more significant response than Mel270 in regard to the ATP reduction in 2D cell cultures, whereas the ATP levels in the spheroids are comparable for both cell lines. The difference between the two tested models could be accredited to a more accurate simulation of morphological and biochemical features of the original tissue cells in the spheroids since 3D cell cultures offer investigative conditions that closely resemble the in vivo situation. In respect of the cell viability, a significant reduction was detected with concentrations of calcium chloride higher than 0.28 mg/mL. For the UM92.1 and Mel270 cell lines, a similarly significant effect regarding cell viability and specific growth rate reduction could be achieved in monolayer cell cultures after ECT with both bleomycin concentrations and following CaEP only after application of 1.1 mg/mL calcium chloride. Analyzing the image documentation of the spheroids, a more evident size reduction and detachment manifest in the UM92.1 and Mel270 cell lines in comparison to the UPM spheroids after both treatment modalities and independently from the administered concentrations. The detachment is more prominent after CaEP in comparison to ECT for both UM92.1 and Mel270, whereas ECT has a lower effect in concern to the spheroid detachment and a higher impact on the spheroid size, especially in the UM92.1 cell line. The low cohesion and peripheral cell distribution seen in the spheroid imaging explains the increase in the mean gray value following ECT in both cell lines. The greater response and the robust growth development of the UM92.1 and Mel270 spheroids may be associated with the origin of the cell lines. Mel270 cells arose from a large recurrent tumor after prior irradiation [41]. The UM92.1 cells were acquired from a large tumor mass that had destroyed the eye and orbit and led to metastases, although this tumor had disomy of chromosome 3 and expressed BAP1. An EIF1AX mutation was detected, which has been associated with the development of metastases in disomy 3 uveal melanomas [43]. The tumor origin also explains the significant reduction in the survival fraction following the application of bleomycin and calcium without electroporation only in the primary cell lines. The morphologic spheroid changes are also illustrated diagrammatically after the calculation of a significant reduction in the cross-sectional area, optic density, and Feret’s diameter for the two cell lines. Image documentation and analysis represent a supplementary instrument for a simple and rapid assessment of therapy efficacy, cytotoxicity, and the spheroid microenvironment, implementing standard biochemical and histological procedures. EP alone had a significant effect on ATP depletion and cell viability in both UM92.1 and Mel270, also leading to an apparent detachment in the spheroid structure, in contrast to the results in the UPM cell lines and spheroids. On the other hand, no significant differences between the tested cell lines were detected in regard to the colony formation capacity and the survival fraction; thus, all four cell lines showed a significant reduction.

Calcium electroporation is postulated to have a limited impact on normal cells compared to malignant cells, as shown in 3D spheroid models and in vivo [51,52,53,54]. Normal cells show a higher resistance to ATP depletion than malignant cells [31,36,52,53,54,56]. The background for this observation may lie within the impact of CaEP on cytoskeleton components, including the activation of ATP-dependent pumps [48], e.g., plasma membrane calcium ATPase (PMCA), sodium calcium exchanger (NCX1) [51] or SERCA2/3 [33,34,35] as well as the altered production of ATP as a reaction to higher intracellular calcium concentrations [32]. The spatial structure of the actin cytoskeleton is crucial for numerous processes such as cell migration, membrane extension, and extracellular signal transduction [57]. A different response to CaEP was documented in normal cells due to a higher expression of PMCA in comparison to malignant cells, which induces the fast removal of calcium ions from the cytosol and could indicate higher viability [54]. One other component that differs between normal and malignant cells in the fraction and localization of the negatively charged phospholipid, phosphatidylserine [58]. Normal cells exhibit plasma membrane asymmetry, and phosphatidylserine is almost exclusively localized in the inner leaflet of the cell membrane unless undergoing apoptosis. Calcium interacts with phosphatidylserine, stabilizing the cell membrane, and the localization of phosphatidylserine in the outer cell membrane leaflet of malignant cells may therefore influence the effect of calcium electroporation [59].

Furthermore, the temperature and the time of administration prior to or after electroporation have been discussed as possible factors with an impact on the efficacy of CaEP [39]. In this regard, EP and ECT combined with concomitant irradiation enhance the radiosensitivity of UM. Our group has previously presented the effect of ECT by altering electric settings in combination with various chemotherapeutic agents in various concentrations [27] in ocular conjunctival and uveal melanoma. Further studies optimizing the EP conditions and calcium concentrations as well as investigating other influencing variables are required to explore the CaEP outcome in vivo for malignant tumors. Calcium displays low cytotoxicity, especially when applied locally, and is therefore only associated with minor adverse effects compared to other cancer treatments or chemotherapeutic agents used in ECT, constituting it a suitable alternative adjuvant therapy for the improvement of local tumor control.

## 5. Conclusions

The present study aims to evaluate the cytotoxicity, the morphological changes of tumor spheroids, the effect on the cell viability, the specific growth rate, and the survival fraction following CaEP compared to ECT in 2D monolayer cell cultures as well as in 3D tumor spheroids in four UM cell lines. A dose-dependent reduction in ATP levels, cell viability, and specific growth rate with varying sensitivity among the tested cell lines was documented. The limitation of our study is the short lifespan of spheroids, which affects the size of the treated tumor as well as the central cell necrosis in the tumor organoids. CaEP is a novel treatment modality, and little knowledge has been generated up to now regarding its pathomechanism, and the optimal therapeutic settings and further dependent variables remain to be explored. The use of 3D spheroid models imitates an in vivo setting with higher precision, delivering more representative results for optimization of CaEP settings for targeted treatment. Our in vitro results indicate that CaEP may constitute an efficient and inexpensive therapeutic option for the local tumor control of UM and the prolongation of life expectancy in patients with progressive disease.

## References

[1] Kaliki S., Shields C.L. (2017). Uveal melanoma: Relatively rare but deadly cancer. Eye.

[2] Shields C.L., Furuta M., Thangappan A., Nagori S., Mashayekhi A., Lally D.R., Kelly C.C., Rudich D.S., Nagori A.V., Wakade O.A. (2009). Metastasis of uveal melanoma millimeter-by-millimeter in 8033 consecutive eyes. Arch. Ophthalmol..

[3] Coupland S.E., Lake S.L., Zeschnigk M., Damato B.E. (2013). Molecular pathology of uveal melanoma. Eye.

[4] Dogrusoz M., Jager M.J., Damato B. (2017). Uveal Melanoma Treatment and Prognostication. Asia Pac. J. Ophthalmol..

[5] Kaliki S., Shields C.L., Shields J.A. (2015). Uveal melanoma: Estimating prognosis. Indian J. Ophthalmol..

[6] Amaro A., Gangemi R., Piaggio F., Angelini G., Barisione G., Ferrini S., Pfeffer U. (2017). The biology of uveal melanoma. Cancer Metastasis Rev..

[7] Helgadottir H., Hoiom V. (2016). The genetics of uveal melanoma: Current insights. Appl. Clin. Genet..

[8] Weis E., Shah C.P., Lajous M., Shields J.A., Shields C.L. (2006). The association between host susceptibility factors and uveal melanoma: A meta-analysis. Arch. Ophthalmol..

[9] Rai K., Pilarski R., Cebulla C.M., Abdel-Rahman M.H. (2016). Comprehensive review of BAP1 tumor predisposition syndrome with report of two new cases. Clin. Genet..

[10] Doherty R.E., Alfawaz M., Francis J., Lijka-Jones B., Sisley K., Scott J.F., Gerstenblith M.R. (2018). Genetics of Uveal Melanoma, in Noncutaneous Melanoma.

[11] Riechardt A.I., Kilic E., Joussen A.M. (2021). The Genetics of Uveal Melanoma: Overview and Clinical Relevance. Klin. Monbl. Augenheilkd.

[12] Chattopadhyay C., Kim D.W., Gombos D.S., Oba J., Qin Y., Williams M.D., Esmaeli B., Grimm E.A., Wargo J.A., Woodman S.E. (2016). Uveal melanoma: From diagnosis to treatment and the science in between. Cancer.

[13] Croce M., Ferrini S., Pfeffer U., Gangemi R. (2019). Targeted Therapy of Uveal Melanoma: Recent Failures and New Perspectives. Cancers.

[14] Triozzi P.L., Eng C., Singh A.D. (2008). Targeted therapy for uveal melanoma. Cancer Treat. Rev..

[15] Singh A.D., Turell M.E., Topham A.K. (2011). Uveal melanoma: Trends in incidence, treatment, and survival. Ophthalmology.

[16] Mallone F., Sacchetti M., Lambiase A., Moramarco A. (2020). Molecular Insights and Emerging Strategies for Treatment of Metastatic Uveal Melanoma. Cancers.

[17] Campana L.G., Valpione S., Mocellin S., Sundararajan R., Granziera E., Sartore L., Chiarion-Sileni V., Rossi C.R. (2012). Electrochemotherapy for disseminated superficial metastases from malignant melanoma. Br. J. Surg..

[18] Heller R., Jaroszeski M.J., Reintgen D.S., Puleo C.A., DeConti R.C., Gilbert R.A., Glass L.F. (1998). Treatment of cutaneous and subcutaneous tumors with electrochemotherapy using intralesional bleomycin. Cancer.

[19] Matthiessen L.W., Chalmers R.L., Sainsbury D.C., Veeramani S., Kessell G., Humphreys A.C., Bond J.E., Muir T., Gehl J. (2011). Management of cutaneous metastases using electrochemotherapy. Acta Oncol..

[20] Quaglino P., Mortera C., Osella-Abate S., Barberis M., Illengo M., Rissone M., Savoia P., Bernengo M.G. (2008). Electrochemotherapy with intravenous bleomycin in the local treatment of skin melanoma metastases. Ann. Surg. Oncol..

[21] Fiorentzis M., Kalirai H., Katopodis P., Seitz B., Viestenz A., Coupland S.E. (2018). Electrochemotherapy with bleomycin and cisplatin enhances cytotoxicity in primary and metastatic uveal melanoma cell lines in vitro. Neoplasma.

[22] Fiorentzis M., Viestenz A., Siebolts U., Seitz B., Coupland S.E., Heinzelmann J. (2019). The Potential Use of Electrochemotherapy in the Treatment of Uveal Melanoma: In Vitro Results in 3D Tumor Cultures and In Vivo Results in a Chick Embryo Model. Cancers.

[23] Larkin J.O., Collins C.G., Aarons S., Tangney M., Whelan M., O’Reily S., Breathnach O., Soden D.M., O’Sullivan G.C. (2007). Electrochemotherapy: Aspects of preclinical development and early clinical experience. Ann. Surg..

[24] Edhemovic I., Gadzijev E.M., Brecelj E., Miklavcic D., Kos B., Zupanic A., Mali B., Jarm T., Pavliha D., Marcan M. (2011). Electrochemotherapy: A new technological approach in treatment of metastases in the liver. Technol. Cancer Res. Treat..

[25] Kranjc S., Cemazar M., Grosel A., Scancar J., Sersa G. (2003). Electroporation of LPB sarcoma cells in vitro and tumors in vivo increases the radiosensitizing effect of cisplatin. Anticancer Res..

[26] Kranjc S., Cemazar M., Grosel A., Sentjurc M., Sersa G. (2005). Radiosensitising effect of electrochemotherapy with bleomycin in LPB sarcoma cells and tumors in mice. BMC Cancer.

[27] Fiorentzis M., Sokolenko E.A., Bechrakis N.E., Ting S., Schmid K.W., Sak A., Stuschke M., Seitz B., Berchner-Pfannschmidt U. (2021). Electrochemotherapy with Bleomycin Enhances Radiosensitivity of Uveal Melanomas: First In Vitro Results in 3D Cultures of Primary Uveal Melanoma Cell Lines. Cancers.

[28] Carafoli E., Santella L., Branca D., Brini M. (2001). Generation, control, and processing of cellular calcium signals. Crit. Rev. Biochem. Mol. Biol..

[29] Berridge M.J., Bootman M.D., Roderick H.L. (2003). Calcium signalling: Dynamics, homeostasis and remodelling. Nat. Rev. Mol. Cell Biol..

[30] Frandsen S.K., Gissel H., Hojman P., Tramm T., Eriksen J., Gehl J. (2012). Direct therapeutic applications of calcium electroporation to effectively induce tumor necrosis. Cancer Res..

[31] Zhivotovsky B., Orrenius S. (2011). Calcium and cell death mechanisms: A perspective from the cell death community. Cell Calcium.

[32] Cerella C., Diederich M., Ghibelli L. (2010). The dual role of calcium as messenger and stressor in cell damage, death, and survival. Int. J. Cell Biol..

[33] Endo Y., Uzawa K., Mochida Y., Shiiba M., Bukawa H., Yokoe H., Tanzawa H. (2004). Sarcoendoplasmic reticulum Ca(2+) ATPase type 2 downregulated in human oral squamous cell carcinoma. Int. J. Cancer.

[34] Bergner A., Kellner J., Tufman A., Huber R.M. (2009). Endoplasmic reticulum Ca2+-homeostasis is altered in Small and non-small Cell Lung Cancer cell lines. J. Exp. Clin. Cancer Res..

[35] Gelebart P., Kovács T., Brouland J.P., van Gorp R., Grossmann J., Rivard N., Panis Y., Martin V., Bredoux R., Enouf J. (2002). Expression of endomembrane calcium pumps in colon and gastric cancer cells. Induction of SERCA3 expression during differentiation. J. Biol. Chem..

[36] Frandsen S.K., Gissel H., Hojman P., Eriksen J., Gehl J. (2014). Calcium electroporation in three cell lines: A comparison of bleomycin and calcium, calcium compounds, and pulsing conditions. Biochim. Biophys. Acta.

[37] Frandsen S.K., Gehl J. (2017). Effect of calcium electroporation in combination with metformin in vivo and correlation between viability and intracellular ATP level after calcium electroporation in vitro. PLoS ONE.

[38] Romeo S., Sannino A., Scarfì M.R., Vernier P.T., Cadossi R., Gehl J., Zeni O. (2018). ESOPE-Equivalent Pulsing Protocols for Calcium Electroporation: An In Vitro Optimization Study on 2 Cancer Cell Models. Technol. Cancer Res. Treat..

[39] Hoejholt K.L., Mužić T., Jensen S.D., Dalgaard L.T., Bilgin M., Nylandsted J., Heimburg T., Frandsen S.K., Gehl J. (2019). Calcium electroporation and electrochemotherapy for cancer treatment: Importance of cell membrane composition investigated by lipidomics, calorimetry and in vitro efficacy. Sci. Rep..

[40] De Waard-Siebinga I., Blom D.J., Griffioen M., Schrier P.I., Hoogendoorn E., Beverstock G., Danen E.H., Jager M.J. (1995). Establishment and characterization of an uveal-melanoma cell line. Int. J. Cancer.

[41] Jager M.J., Magner J.A., Ksander B.R., Dubovy S.R. (2016). Uveal Melanoma Cell Lines: Where do they come from? (An American Ophthalmological Society Thesis). Trans. Am. Ophthalmol. Soc..

[42] Griewank K.G., Yu X., Khalili J., Sozen M.M., Stempke-Hale K., Bernatchez C., Wardell S., Bastian B.C., Woodman S.E. (2012). Genetic and molecular characterization of uveal melanoma cell lines. Pigment Cell Melanoma Res..

[43] Amirouchene-Angelozzi N., Nemati F., Gentien D., Nicolas A., Dumont A., Carita G., Camonis J., Desjardins L., Cassoux N., Piperno-Neumann S. (2014). Establishment of novel cell lines recapitulating the genetic landscape of uveal melanoma and preclinical validation of mTOR as a therapeutic target. Mol. Oncol..

[44] Van den Aardweg G.J., Kiliç E., de Klein A., Luyten G.P. (2003). Dose fractionation effects in primary and metastatic human uveal melanoma cell lines. Invest. Ophthalmol. Vis. Sci..

[45] Nareyeck G., Zeschnigk M., Bornfeld N., Anastassiou G. (2009). Novel cell lines derived by long-term culture of primary uveal melanomas. Ophthalmologica.

[46] Falk H., Matthiessen L.W., Wooler G., Gehl J. (2018). Calcium electroporation for treatment of cutaneous metastases; a randomized double-blinded phase II study, comparing the effect of calcium electroporation with electrochemotherapy. Acta Oncol..

[47] Novickij V., Čėsna R., Perminaitė E., Zinkevičienė A., Characiejus D., Novickij J., Šatkauskas S., Ruzgys P., Girkontaitė I. (2019). Antitumor Response and Immunomodulatory Effects of Sub-Microsecond Irreversible Electroporation and Its Combination with Calcium Electroporation. Cancers.

[48] Gibot L., Montigny A., Baaziz H., Fourquaux I., Audebert M., Rols M.P. (2020). Calcium Delivery by Electroporation Induces In Vitro Cell Death through Mitochondrial Dysfunction without DNA Damages. Cancers.

[49] Clapham D.E. (2007). Calcium signaling. Cell.

[50] Furuya Y., Lundmo P., Short A.D., Gill D.L., Isaacs J.T. (1994). The role of calcium, pH, and cell proliferation in the programmed (apoptotic) death of androgen-independent prostatic cancer cells induced by thapsigargin. Cancer Res..

[51] Szewczyk A., Gehl J., Daczewska M., Saczko J., Frandsen S.K., Kulbacka J. (2018). Calcium electroporation for treatment of sarcoma in preclinical studies. Oncotarget.

[52] Frandsen S.K., Gibot L., Madi M., Gehl J., Rols M.P. (2015). Calcium Electroporation: Evidence for Differential Effects in Normal and Malignant Cell Lines, Evaluated in a 3D Spheroid Model. PLoS ONE.

[53] Zielichowska A., Daczewska M., Saczko J., Michel O., Kulbacka J. (2016). Applications of calcium electroporation to effective apoptosis induction in fibrosarcoma cells and stimulation of normal muscle cells. Bioelectrochemistry.

[54] Frandsen S.K., Krüger M.B., Mangalanathan U.M., Tramm T., Mahmood F., Novak I., Gehl J. (2017). Normal and Malignant Cells Exhibit Differential Responses to Calcium Electroporation. Cancer Res..

[55] Ewens K.G., Kanetsky P.A., Richards-Yutz J., Purrazzella J., Shields C.L., Ganguly T., Ganguly A. (2014). Chromosome 3 status combined with BAP1 and EIF1AX mutation profiles are associated with metastasis in uveal melanoma. Investig. Ophthalmol. Vis. Sci..

[56] Hansen E.L., Sozer E.B., Romeo S., Frandsen S.K., Vernier P.T., Gehl J. (2015). Dose-dependent ATP depletion and cancer cell death following calcium electroporation, relative effect of calcium concentration and electric field strength. PLoS ONE.

[57] Drees B.E., Andrews K.M., Beckerle M.C. (1999). Molecular dissection of zyxin function reveals its involvement in cell motility. J. Cell Biol..

[58] Sharma B., Kanwar S.S. (2018). Phosphatidylserine: A cancer cell targeting biomarker. Semin. Cancer Biol..

[59] Levine Z.A., Vernier P.T. (2012). Calcium and phosphatidylserine inhibit lipid electropore formation and reduce pore lifetime. J. Membr. Biol..

